# Long non-coding RNAs as the critical regulators of epithelial mesenchymal transition in colorectal tumor cells: an overview

**DOI:** 10.1186/s12935-022-02501-5

**Published:** 2022-02-10

**Authors:** Amir Abbas Hamidi, Ghazaleh Khalili-Tanha, Zahra Nasrpour Navaei, Meysam Moghbeli

**Affiliations:** 1 Student Research Committee, Faculty of Medicine, Mashhad University of Medical Sciences, Mashhad, Iran; 2 Department of Medical Genetics and Molecular Medicine, School of Medicine, Mashhad University of Medical Sciences, Mashhad, Iran

**Keywords:** Colorectal cancer, Metastasis, EMT, lncRNA, Diagnosis, Prognosis

## Abstract

Colorectal cancer (CRC) is the second most common cause of cancer mortality and a major health challenge worldwide. Despite advances in therapeutic and diagnostic methods, there is still a poor prognosis in CRC patients. Tumor recurrence and metastasis are the main causes of high mortality rate in these patients, which are due to late diagnosis in advanced tumor stages. Epithelial-mesenchymal transition (EMT) is known to be the most important cause of CRC metastasis, during which tumor cells obtain metastasis ability by losing epithelial features and gaining mesenchymal features. Long non-coding RNAs (lncRNAs) are pivotal regulators of EMT process. Regarding the higher stability of lncRNAs compared with coding RNAs in body fluids, they can be used as non-invasive diagnostic markers for EMT process. In the present review, we summarized all of the lncRNAs involved in regulation of EMT process during CRC progression and metastasis. It was observed that lncRNAs mainly induced the EMT process in CRC cells by regulation of EMT-related transcription factors, Poly comb repressive complex (PRC), and also signaling pathways such as WNT, NOTCH, MAPK, and Hippo.

## Background

Colorectal cancer (CRC) is the second most common cause of cancer-related deaths globally [1]. In spite of improvements in therapeutic methods, there is still a low overall survival rate in CRC patients [2, 3]. The incidence of recurrence and distant metastasis in late-stage CRC is the most plausible explanation for reduced survival rates [4]. Advanced-stage CRC patients have a poor prognosis, regardless of advancements in surgical procedures and adjuvant chemo-radio therapeutic treatments [5, 6]. Colon cancer initiation and development is an intricate, multi-step, and multifactorial process associated with genetic and epigenetic modifications [7]. CRC poses health and economic difficulties for countries due to its high death rate and poor prognosis. The majority of CRC cases are observed in advanced stages with metastasis that is a big therapeutic challenge. Therefore, additional researches are required about the molecular mechanisms of CRC initiation and progression to detect novel diagnostic and prognostic biomarkers. Epithelial-mesenchymal transition (EMT) has a crucial role in the pathogenesis of CRC [8]. Upon the activation of EMT, CRC cells may spread to adjacent tissues or distant organs via the vascular system [9, 10]. EMT process is characterized as the transformation of epithelial cells into mesenchymal cells by loss of the cell–cell adhesion. This process is critical for embryogenesis, tumor growth, tissue repair, and fibrotic scarring [11, 12]. TWIST, ZEB1, ZEB2, SNAIL1, and SNAIL2 are some of the main transcription factors that promote EMT progression [11, 13]. During EMT, epithelial cells should remove extracellular restrictions including cell adhesion molecules to gain increased migratory and invasive properties [14, 15]. E-cadherin, as a Ca2 + -dependent adhesion molecule maintains the stability of cell–cell connections [16]. Epithelial cells connections rely on adherence junctions and desmosomes on the lateral side and tight junctions on the apical side. Tight junctions are formed by occludin and claudin. The adherence junction is also a structure made up of cell adhesion molecules (E-cadherin and nectin) bound to the cytoskeleton through afadin and catenin. In epithelial cells, EMT results in loss of cell polarity following the E-cadherin, claudin, and occludin down regulations. EMT can also stimulate the MMP up regulation, which is involved in the degradation and destruction of extracellular matrix (collagen, laminin, and fibronectin) and basal membrane. Destroying the tissue barrier of tumor cells promote the cells to separate from primary tumor and metastasize. MicroRNAs (miRNAs), long non-coding RNAs (lncRNAs), transfer RNA (tRNA) fragments, and long enhancer ncRNAs (eRNAs) are among the various classes of non-coding RNAs [17–20]. LncRNAs are involved in cell differentiation, proliferation, apoptosis, epigenetic processes and miRNA regulation by pre-transcriptional, transcriptional, and post-transcriptional levels regulations [21]. The most widely described molecular mechanism underpinning lncRNA-mediated tumorigenesis is that they operate as competing endogenous RNA (ceRNA) to sponge microRNAs, resulting in the positive regulation of miRNA target genes [22–24]. LncRNAs have pivotal roles in CRC initiation and progression by acting as oncogenes or tumor suppressor genes [25]. Down regulation of various lncRNAs such as SPRY4-IT1, Linc01194, ADAMTS9-AS1, lncRNA-ATB, and AGAP2-AS1 also up regulate E-cadherin expression while significantly decrease Vimentin expression in CRC cells [26–30]. In the present review, we have summarized and discussed all of the lncRNAs associated with EMT regulation during CRC progression (until July 2021) (Table 1).

**Table1:** Role of lncRNAs in EMT regulation in colorectal tumor cells

LncRNA	Target	Samples	Function	Year	Refs.
H19	miR-29b-3p/ PGRN	185 NT* HCT116, HT29, SW620, and SW480 cell lines	Induced EMT	2018	Ding et al. [22]
AGAP2-AS1	miR-4668-3p/ SRSF1	SW620, HT-29, and HCT8 cell lines	Induced EMT	2020	Li et al. [26]
ATB	CDH1	60 NT, 50 patients’ blood, 50 control blood SW480, HCT116, CACO2, CACO205, SW620, and LOVO cell lines	Induced EMT	2016	Yue et al. [27]
ADAMTS9-AS1	-	59 NT SW116, RKO, HT29, and HCT116 cell lines	Induced EMT	2020	Chen et al. [28]
LINC01194	CDH2/ VIM	467 NT HT29, HCT116, and SW620 cell lines	Induced EMT	2019	Wang et al. [29]
SLCO4A1-AS1	CTNNB1/ GSKb	50 NT HCT116, HCT8, HT29, LOVO, and SW620 cell lines	Induced EMT	2018	Yu et al. [44]
CYTOR	CTNNB1	100 NT HCT116, SW1417, SW620, HT29, CACO2, SW480, and HCT8 cell lines	Induced EMT	2018	Yue et al. [45]
LINC01354	hnRNP-D/ WNT	88 NT HCT116, HT29, LOVO, SW620, and SW480 cell lines	Induced EMT	2019	Li et al. [46]
CTD903	WNT	115 NT RKO, SW480, SW620, DLD1, CACO2, HCT116, and HT29 cell lines	Suppressed EMT	2016	Yuan et al. [48]
ARAP-AS1	WNT	82 NT SW620, SW480, HT29, LOVO, and HCT116 cell lines	Induced EMT	2019	Ye et al. [49]
FOXD2-AS1	NOTCH	45 NT RKO, HCT15, HCT28, HCT116, and SW480 cell lines	Induced EMT	2017	Yang et al. [63]
DSCAM-AS1	miR-137/ NOTCH1	51 NT HT29, LOVO, PKO, and SW480 cell lines	Induced EMT	2020	Xu et al. [66]
SLC25A25-AS1	ERK/ P38	30 NT HCT116 and HT29 cell lines	Suppressed EMT	2016	Li et al. [79]
TUG1	miR-26a-5p/ MMP14/ P38/ HSP27	SW620, HT29, and CACO2 cell lines	Induced EMT	2019	Tian et al. [82]
NNT-AS1	MAPK/ ERK	70 NT SW480 and SW620 cell lines	Induced EMT	2017	Wang et al. [83]
H19	hnRNPA2B1	60 NT HCT116, SW480, and DLD1	Induced EMT	2020	Zhang et al. [85]
SNHG16	miR-124-3p/ MCP1	120 NT SW480, DLD-1, LOVO, and HCT116 cell lines	Induced EMT	2020	Chen et al. [89]
TUG1	TWIST1	27 NT LOVO, HT29, and HCT116 cell lines	Induced EMT	2020	Shen et al. [91]
SNHG6	UPF1/ ZEB1	77 NT HT29, CACO2, SW480, RKO, SW620, LOVO, and HCT116 cell lines	Induced EMT	2019	Wang et al. [93]
SNHG14	miR-32-5p/ SKIL	LoVo, RKO, SW480, and HT-29 cell lines	Induced EMT	2019	Ye et al. [94]
PVT1	miR-216a-5p/ YBX1	70 NT LOVO, SW480, HT29, HCT116, and CACO2 cell lines	Induced EMT	2019	Zeng et al. [98]
CASC21	miR-7-5p/ YAP1	HT29 and SW480 cell lines	Induced EMT	2020	Zheng et al. [108]
MIR4435-2HG	miR-206/ YAP1	90 NT HT-29, SW620, LoVo, and HCT116 cell lines	Induced EMT	2020	Dong et al. [109]
LINC00460	WWC2	62 NT HCT15, HCT116, SW480, SW620, RKO, LOVO, and HT29 cell lines	Induced EMT	2020	Yuan et al. [113]
MALAT1	EZH2	68 NT HT29, SW480, and SW620 cell lines	Induced EMT	2017	Li et al. [120]
SNHG14	EZH2/EPHA7	LOVO,SW620, SW480, HCT116, and HT29 cell lines	Induced EMT	2019	Di et al. [123]
XIST	miR-137/ EZH2	20 NT LOVO, HT29, and SW620 cell lines	Induced EMT	2018	Liu et al. [130]
B3GALT5-AS1	miR-203/ ZEB2	64 NT HCT116, HT29, LOVO, SW480, and SW620 cell lines	Induced EMT	2018	Wang et al. [135]
HOTAIR	HNF4a	124 NT HCT116 and HT29 cell lines	Induced EMT	2021	Jin et al. [139]
HOTAIR	CDH1	120 NT HT29, SW480, SW620, RKO, HCT116, and LOVO cell lines	Suppressed EMT	2014	Wu et al. [140]
HOXA11-AS1	miR-149-3p	105 NT HCT116 cell line	Induced EMT	2020	Chen et al. [141]
EWSAT1	CDH1	106 NT SW480, HT29, and SW620 cell lines	Induced EMT	2018	Zhang et al. [143]
LDLRAD4-AS1	LDLRAD4/ SNAIL	RKO, LOVO, DLD1, HCT116, HCT8, and HT29 cell lines	Induced EMT	2020	Mo et al. [144]
CHRF	miR-489/ TWIST1	80 NT HCT116 and SW480 cell lines	Induced EMT	2017	Tao et al. [145]
KCNQOT1	miR-217/ ZEB1	HT29, HCT116, SW480, and DLD1 cell lines	Induced EMT	2019	Bian et al. [154]
ZFAS1	ZEB1	73 NT, 105 patients’ blood, 95 control blood SW620 cell line	Induced EMT	2017	Fang et al. [155]
XIST	miR-200b-3p/ ZEB1	115 NT HCT116, HT29, SW620, RKO, SW480, and LOVO cell lines	Induced EMT	2017	Chen et al. [156]
XIST	miR-125b-2-3p	122 NT DLD1, HCT116, and HCT8 cell lines	Induced EMT	2021	Zeng et al. [157]
TUG1	miR-138-5p/ ZEB2	84 NT LOVO cell line	Induced EMT	2020	Yan et al. [158]
UICLM	miR-215/ ZEB2	SW620, SW480, LOVO, HT29, HCT116, RKO, and DLD1 cell lines	Induced EMT	2017	Chen et al. [161]
CRCMSL	HMGB2	HCT116, SW480, SW620, HT29, and LOVO cell lines	Suppressed EMT	2019	Han et al. [165]
CPS1-IT1	HIF-1α	24 NT LoVo, SW620, SW480, LS174T, HCT116, and HT29 cell lines	Suppressed EMT	2017	Zhang et al. [173]
XIST	miR-93-5p/ HIF1a	36 NT SW480 and LOVO cell lines	Induced EMT	2020	Yang et al. [174]

^*^Normal (N) and Tumor (T) tissues

## WNT and NOTCH signaling pathways

**Fig. 1:**
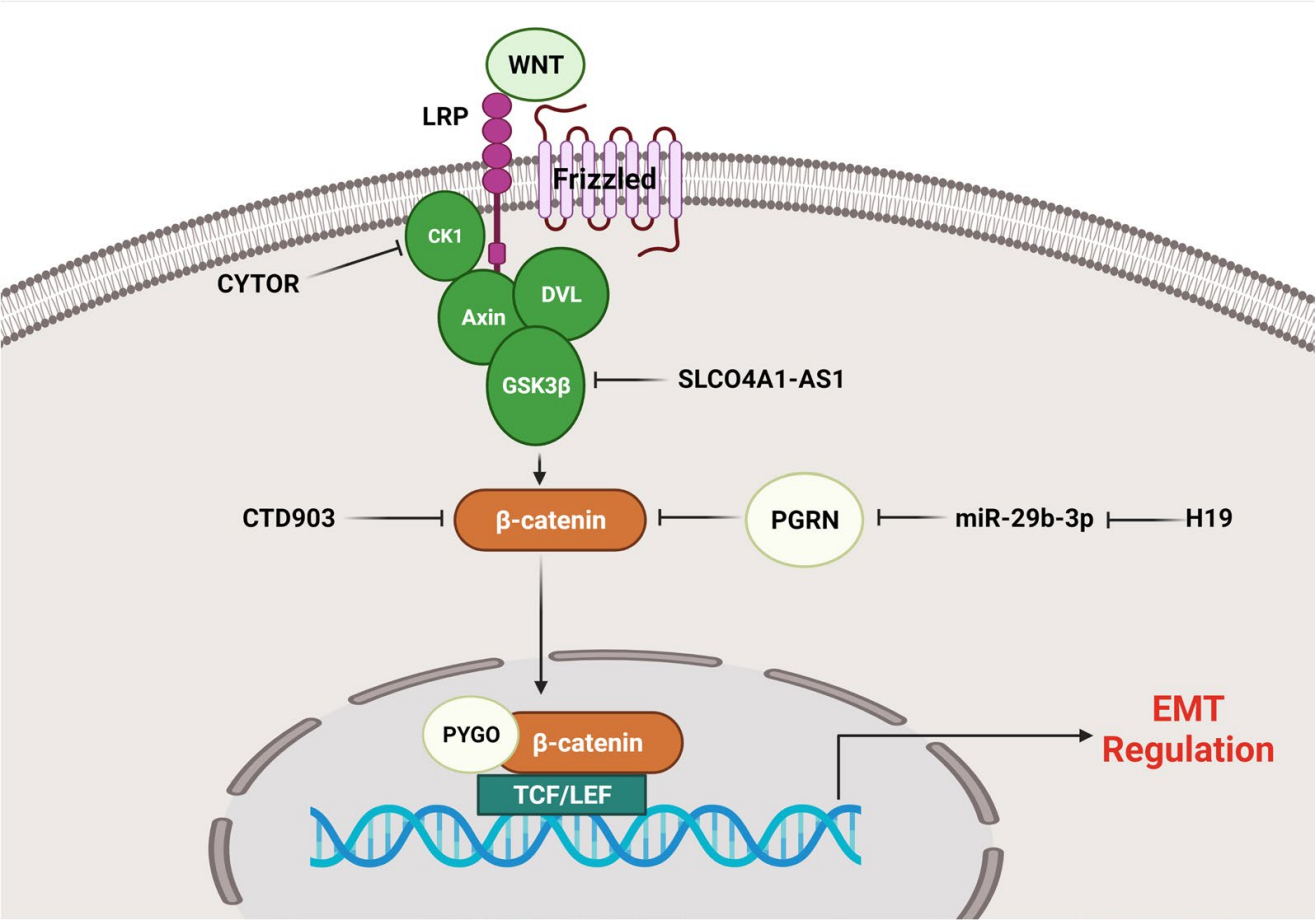
Role of lncRNAs in EMT regulation via Wnt/β-catenin signaling pathway in CRC. CYTOR inhibited CK1-dependent phosphorylation of cytoplasmic β-catenin, promoting nuclear translocation of β-catenin. By suppressing GSKβ-mediated phosphorylation, SLCO4A1-AS1 increased the stability of β-catenin. CTD903 suppressed cell invasion, migration, EMT, and Wnt/β-catenin signaling through down regulation of β-catenin. (Created with www.BioRender.com)

WNT signaling pathway is involved in various cellular processes such as embryogenesis, cell proliferation, differentiation, and migration [31–33]. Frizzled and low-density lipoprotein receptor-related protein (LRP) are cell membrane receptors that transmit WNT signaling across the plasma membrane. B-catenin is phosphorylated by the complex of GSK-3β, Axin, and adenomatous polyposis coli (APC) in the lack of WNT signaling, leading to the sequestration of cytoplasmic β-catenin and its proteasomal degradation [34]. Following the stimulation of Frizzled by Wnt ligands, GSK-3β phosphorylates LPR6, and meanwhile, Dishevelled (DVL) and Axin are recruited to the plasma membrane. The activation of Frizzled receptors by Wnt ligands also enhances the DVL-mediated suppression of GSK-3β. Repressed GSK-3β is incapable of forming a complex with Axin and thus cannot phosphorylate β-catenin, resulting in the translocation of β-catenin into the nucleus. Nuclear β-catenin binds to the TCF/LEF transcription factors to induce EMT. Cytoplasmic GSK-3β accumulation, followed by Wnt–β-catenin stimulation, also inhibits the phosphorylation of Snail1, resulting in Snail1 stabilization [35]. Wnt-induced EMT via Snail2 is associated with downregulation of E-cadherin and upregulation of fibronectin, similar to the findings in nuclear β-catenin accumulation [36, 37]. Wnt signaling is abnormally upregulated [38] and directly stimulates the expression of SNAI1 and SNAI2 in various cancers [39]. Wnt has also been found to be correlated with upregulation of TWIST expression in epithelial cells, which is one of the key inducers of EMT [40]. LncRNAs have pivotal roles in EMT regulation in CRC via Wnt/β-catenin signaling pathway (Fig. 1). β-catenin is a critical component of Wnt signaling that can modulate the expression of many transcription factors to trigger EMT process [41–43]. SLCO4A1-AS1 up regulation was associated with a poor prognosis and malignant tumor in CRC patients. SLCO4A1-AS1 knock down reduced CRC cells invasion and EMT by CDH1 up regulation and Vimentin down regulation. By suppressing GSKβ-mediated phosphorylation, SLCO4A1-AS1 increased the stability of β-catenin. SLCO4A1-AS1 prevented β-catenin from being phosphorylated and being degraded by ubiquitin. SLCO4A1-AS1 disrupted the interaction of GSK3β with β-catenin by binding to β-catenin that suppressed GSK3β-mediated β-catenin phosphorylation [44]. It has been observed that CYTOR promoted EMT and metastatic behavior in CRC. CYTOR inhibited CK1-dependent phosphorylation of cytoplasmic β-catenin, promoting nuclear translocation of β-catenin. Moreover, β-catenin stimulated CYTOR transcription in the nucleus in a reciprocal manner, resulting in a feed-forward loop [45]. LINC01354 knock down impaired CRC cell growth and metastasis. LINC01354 interacted with hnRNP-D to activate Wnt/β-catenin signaling pathway in CRC cells. LINC01354 expression levels were correlated with lymph node involvement, tumor size, tumor stage, and distant metastasis. It promoted CRC cell proliferation, migration, and EMT process by regulation of β-catenin stability and Wnt/β-catenin pathway activation [46]. LINC00339 was highly expressed in colon cancer cells and tissues. It sponged miR-378a-3p to regulate MED19 that resulted in promotion of WNT signaling pathway. There was a correlation between LINC00339 up regulation and tumor stage in CRC patients. LINC00339 increased N-cadherin and vimentin expressions, while down regulated CDH1. LINC00339 enhanced cell growth, migration, and EMT in CRC via stimulating the Wnt/β-catenin signaling cascade [47]. It has been reported that H19 up regulation was associated with initiation of tumor cell metastasis and EMT. MiR-29b-3p could directly bind to PGRN, altering downstream Wnt signaling and thereby significantly regulating the EMT process. H19/miR-29-3b/PGRN/ Wnt signaling facilitated the initiation of EMT in CRC [22]. CTD903 up regulation was correlated with reduced tumor size, fewer mucinous adenocarcinomas, and a better outcome in CRC cancer. Tumors with elevated CTD903 expression exhibited lower invasive properties than under expressed tumors. It suppressed cell invasion, migration, EMT, and Wnt/β-catenin signaling through down regulation of β-catenin, Twist, and Snail in CRC [48]. YY1/ARAP1-AS1 axis also enhanced colon cancer cell migratory and invasive capacities, as well as EMT progression via the Wnt/b-catenin pathway [49].

Notch signaling pathway is a highly maintained cellular process in different species with important roles in cell cycle, differentiation, apoptosis, angiogenesis, hematopoiesis, neurogenesis and proliferation [50–52]. It needs cell-to-cell contact for activation and consists of multiple receptors and ligands [53]. There are four types of Notch receptors including Notch1-4 that can be activated by Delta/Serrate/Lag2 (DSL) ligands [53–55]. Following the ligand binding, intracellular domain of the notch protein (NICD) is transferred to the nucleus and then regulates expression of downstream genes by forming a complex with CSL and MAML transcriptional regulators. The targeted genes in canonical Notch signaling are Hes1, Hes5, Hes6, Hes7, Hey1, and Hey2 [55]. Notch signaling pathway is involved in CRC initiation, progression, metastasis, and EMT [56, 57]. Notch signaling can, directly [58, 59] and indirectly, modulate SNAI1 expression level by inducing hypoxiα-inducible factor 1α (HIF-1α). Notch is involved in E-cadherin downregulation and β-catenin upregulation by interacting with Snail2 [60, 61]. Elevated expression of Notch in endothelial cells leads to the downregulation of vascular endothelial cadherins, resulting in endothelial to mesenchymal transition [58]. Notch inhibition impairs the capability of NF-κB to bind to DNA and downregulates MMP9, a critical MMP implicated in extracellular matrix remodeling, resulting in increased pancreatic cancer cell extravasation [62]. FOXD2-AS1 expression was considerably deregulated in CRC tissues in comparison with normal tissues. FOXD2-AS1 knockdown inhibited CRC cell proliferative, migratory, and invasive abilities. FOXD2-AS1 may have a role in CRC progression through modulating EMT and the Notch signaling pathway [63]. DSCAM-AS1 deregulation has been reported in a variety of human malignancies [64, 65]. There were significant DSCAM-AS1 up regulations in CRC tissues and cell lines which was positively associated with advanced stage and metastatic status. DSCAM-AS1 expression was associated with poor outcomes in CRC patients. DSCAM-AS1 increased the levels of Notch-1 expressions by miR-137 sponging that promoted CRC cell proliferation, migration, and EMT [66].

## MAPK and TGF-β signaling pathways

**Fig. 2:**
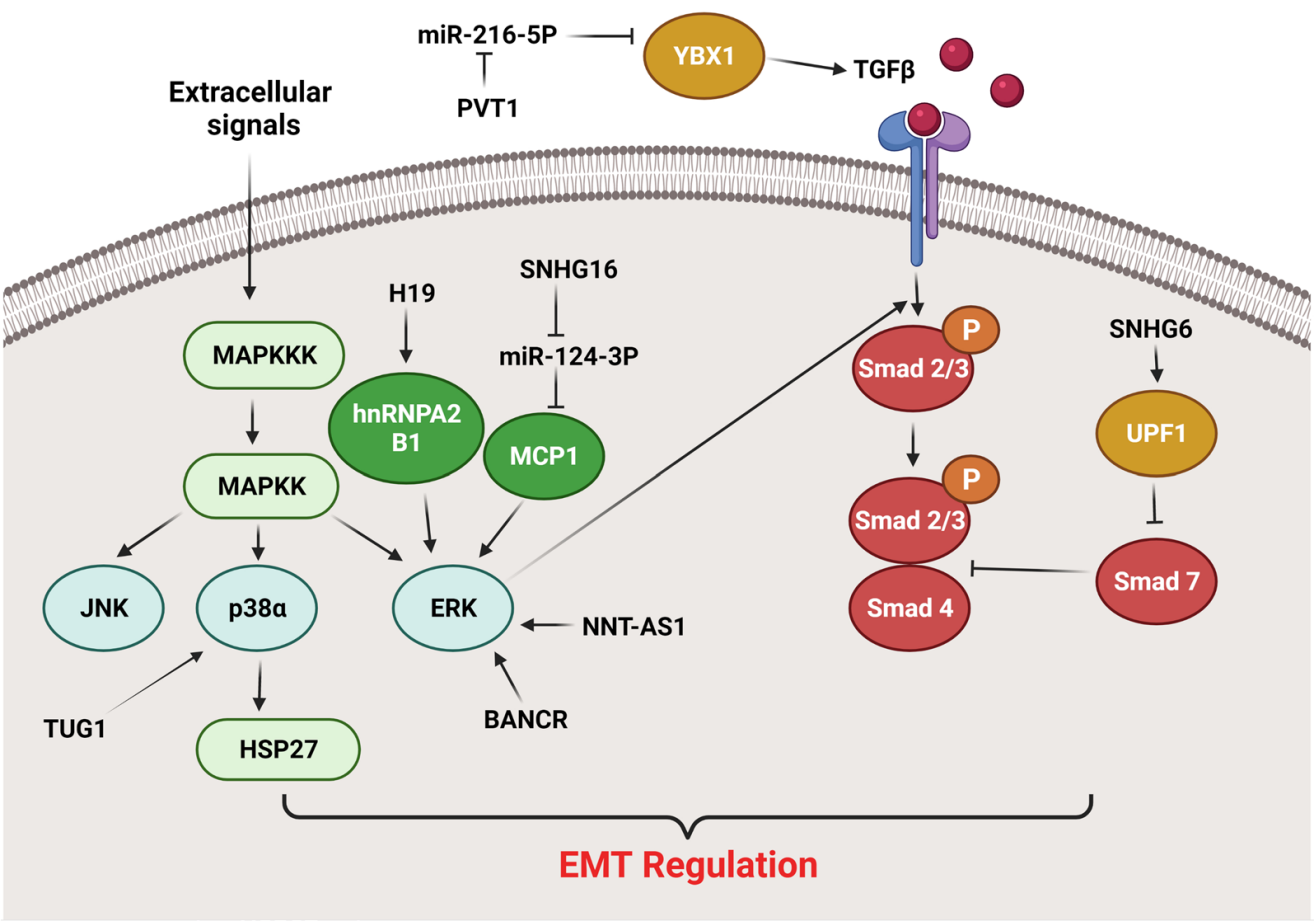
Role of lncRNAs in EMT regulation via MAPK and TGFβ signaling pathways in CRC. PVT1 suppressed the growth and metastatic ability of cancer cells via miR-216a-5p sponging that resulted in YBX1 up regulation. SNHG16 regulated CRC cell proliferation, migration, invasion, and EMT via the miR-124-3p/MCP-1 axis. SNHG6 activated TGF-β/Smad signaling by binding UPF1 that increased tumor cell proliferation and metastasis. TUG1 promoted carcinogenesis and EMT in colon cancer by stimulating the P38MAPK/Hsp27 axis. BANCR promoted CRC cell migration by EMT induction through the MEK/ERK pathway. NNT-AS1 regulated tumor cell proliferation and invasion by reducing the activity of MAPK/ERK pathway to suppress EMT. (Created with www.BioRender.com)

MAPK pathway has pivotal roles in cell migration, growth, apoptosis, and differentiation. The central components of this signaling pathway are ERK1/2, JNK, p38 MAPK, and ERK5. Among them JNK and p38 MAPK are activated by chemical, physical, and biological stimuli while ERK1/2 are activated by cell growth factors [67]. Tyrosine kinase receptors are the main receptors involved in regulation of MAPK signaling pathway. After receptor activation, RAS recruits RAF to phosphorylate MAPK which subsequently activates ERK1/2. ERK1/2 in nucleus activate several transcription factors such as MNK1, Elk-1, and c-Ets1 [68]. The MAPK and STAT3 pathways have been found to be up regulated in a variety of malignancies [69, 70]. MAPK and TGF-β signaling pathways have been shown to interact mutually and synergistically affect the secretion of extra growth factors and cytokines, leading to EMT progression. JNK and p38 MAPK are involved in the TGF-β-induced EMT [71]. After TGF-*β* binds to the receptor extracellular domain, the intracellular domain phosphorylates Smad2/3 and binds to TRAF6, which recruits TAK1 and TAB1 to stimulate JNK and p38 MAPK. Upregulated JNK and p38 MAPK can promote EMT via regulating downstream transcriptional factors in a Smad-dependent or -independent pathway [72]. TRAF6 knockdown inhibits TGF-β-mediated EMT, implying that activation of the TRAF6/TAK1-/p38 MAPK signaling cascade is also required for TGF-β-mediated EMT [73]. ERK activation may play a critical role in various major characteristics of EMT, including the loss of epithelial features and the acquisition of mesenchymal qualities [74]. Research has shown that ERKs suppress E-cadherin expression to promote EMT [75]. TGF-β1-stimulated Slug expression was found to be significantly suppressed by MEK and JNK inhibitors, implying that MAPK pathways are implicated in regulating TGF-β1-stimulated Slug expression [76]. Overall, these findings indicate that the MAPK signaling pathway has a central role in TGF-β-stimulated alterations in actin cytoskeleton structure and cell morphology during EMT. LncRNAs have pivotal roles in EMT regulation in CRC via MAPK and TGF-β signaling pathways (Fig. 2). Solute carrier family 25 member 25(SLC25A25) encodes a Ca2 + -regulated mitochondrial carrier (CaMC) protein which is one of the members of mitochondrial inner membrane protein family [77, 78]. SLC25A25-AS1 is transcribed in the opposite direction of SLC25A25 gene. It has been reported that there were significant SLC25A25-AS1 down regulations in tumor tissues and serum of CRC patients. SLC25A25-AS1 suppressed the proliferative and colony-forming abilities of CRC cell lines. Moreover, SLC25A25-AS1 down regulation increased chemo resistance and induced EMT in vitro via activating the ERK and P38 signaling pathways. Down regulation of SLC25A25-AS1 markedly increased 5-FU or DOX resistance in HT-29 cells [79]. As a trans membrane protein hydrolase, matrix metalloproteinase-14 (MMP-14) degrades cytokines and growth factors concurrently [80]. MMP-14 has been found to increase angiogenesis and tumor metastasis primarily via up regulating VEGF and facilitating MMP-2 activation [81]. Heat shock protein 27 (Hsp27) is regulated by MAPK-activated protein kinase-2 (MAPKAPK2) that is the leading substrate of p38 MAPK. Increased expression of TUG1 has been shown to enhance CRC cell proliferation, invasion, and EMT. TUG1 promoted SW620 cell proliferation and motility in vitro by down regulating miR-26a-5p and up regulating MMP-14. TUG1 modulated carcinogenesis and metastasis through the miR-26a-5p/MMP-14 axis. Moreover, TUG1 promoted carcinogenesis and EMT in colon cancer by stimulating the P38MAPK/Hsp27 axis [82]. There was NNT-AS1 up regulation in CRC tissues in comparison with normal margins that was correlated with lower overall survival. NNT-AS1 knockdown substantially reduced tumor cell proliferation and invasion by reducing the activity of MAPK/ERK pathway to suppress EMT. NNT-AS1 down regulation also reduced vimentin expression whereas increased E-cadherin expression [83]. There were BANCR up regulations in CRC tissues in comparison with normal tissues that were correlated with lymph node metastasis and TNM stage. The expression of epithelial and mesenchymal markers was affected by BANCR expression, suggesting that BANCR may play a role in EMT regulation. BANCR promoted CRC cell migration by EMT induction through the MEK/ERK pathway [84]. H19 and hnRNPA2B1 interaction promoted EMT through the Raf-ERK-dependent pathway that induced CRC progression. There was also H19 up regulation in hepatic metastases compared with primary tumors. Furthermore, H19 up regulation was correlated with lymph node and distant metastases, and poor survival rates. H19 enhanced Snail and N-cadherin expressions, while reduced CDH1 expression levels that triggered EMT and CRC metastasis [85]. Tumor metastasis is a multi-stage process marked by increased invasion, migration, and transition from epithelial to mesenchymal cells. Monocyte chemoattractant protein 1 (MCP-1) is a chemokine implicated in tumor initiation, progression, and metastasis [86, 87]. It promotes EMT process through regulation of ERK/Snail axis [88]. SNHG16 was significantly up regulated in CRC tissues and cell lines. There was also a positive correlation between SNHG16 level and CRC grade. Moreover, SNHG16 knock down inhibited CRC cell proliferation, migration, invasion, and EMT via the miR-124-3p/MCP-1 axis [89].

Transforming growth factor-Beta (TGF-β) signaling is an important pathway involved in cell differentiation, proliferation, apoptosis, and embryogenesis. The TGF-β family of ligands such as Bone morphogenetic proteins (BMPs), Activin, and TGFβs can interact with type I and type II kinase receptors to activate Smad proteins. Smad family proteins includes; receptor-regulated Smad (R-Smad), inhibitory Smad (I-Smad), and the Co-mediator Smad (Co-Smad). After receptor-ligand interaction, the R-Smad proteins make heteromeric complex with Co-Smad proteins. Among ligands, BMPs include BMP2 and 4 have high affinity for receptor I and TGF-β and Activin have high affinity for receptor II. In nucleus both Co-Smads and R-Smads except Smad2 bind to Smad binding element (SBE) on DNA to regulate the target genes. The molecular investigations show relationship between the TGF-β pathway and RAS/MAPK/ERK signaling during development and oncogenesis. The effects of TGF-β pathway on cells depend on cell-type and its link with other pathways [90]. TGF-β, as a key inducer of EMT, stimulates multiple transcriptional regulators, including TWIST1-2, ZEB1-2, and Snai1-2 that results in E-cadherin down regulation. TGF-β has been demonstrated to increase TUG1 expression and CRC cell migration. TUG1 knockdown decreased CRC lung metastasis and inhibited CRC cell invasion and EMT. TGF-β enhanced CRC metastasis through the TUG1/TWIST1/EMT signaling cascade [91]. UPF1 is a component of the nonsense-mediated mRNA decay (NMD) pathway that induces the RNA decay process and destabilize Smad7 to promote TGF-β signaling [92]. It has been shown that SNHG6 activated TGF-β/Smad signaling by binding UPF1 that increased tumor cell proliferation and metastasis. SNHG6 may also modulate ZEB1 by triggering EMT through miR-101-3p targeting [93]. SKIL is a member of SMAD signaling pathway that is involved in regulation of cell growth and differentiation via TGF-β. SNHG14 promoted CRC cell proliferation, metastasis, and EMT through miR-32-5p/SKIL axis [94]. Y-box binding protein 1 (YBX1) is an oncoprotein involved in tumor cell survival, proliferation, therapeutic resistance, chromatin instability, and metastasis [95]. Elevated levels of YBX1 were correlated with local recurrence and overall survival in CRC patients [96]. Moreover, YBX1 acts as a crucial inducer of EMT by increasing the secretion of angiogenic factors, including CSF-1 and TGF-β [97]. There was miR-216a-5p down regulation in CRC patients, which was associated with poor prognosis. MiR-216a-5p significantly suppressed the ability of CRC cell lines to proliferate, migrate, and invade. MiR-216a-5p expression was significantly correlated with lymph node involvement and tumor stage. It also enhanced E-cadherin expression while decreased N-cadherin, Snail, and Vimentin expressions. Therefore, miR216a-5p was proposed to have an anti-metastatic effect in colorectal cancer through EMT regulation. PVT1 suppressed the growth and metastatic ability of cancer cells via miR-216a-5p sponging that resulted in YBX1 up regulation [98]. LINC01133 was observed to suppress EMT and metastasis via binding to SRSF6 in CRC cells. LINC01133 was down regulated by TGF-β that reduced the migratory and invasive capacity of CRC cells. There was also significant LINC01133 down regulation in CRC samples compared with normal margins [99].

## Hippo signaling pathway

Hippo signaling has mainly anti-tumor roles during tumor progression [100]. It is characterized by phosphorylation of YAP1 and TAZ1 [101]. In the absence of an active Hippo signaling pathway, unphosphorylated Yap and Taz enter the nucleus, interact with Tead transcription factors, and stimulate genes involved in proliferation and anti-apoptotic processes [102]. EMT related genes involved in cell–cell adhesion and actin cytoskeleton are upregulated by Tead2 [103]. YAP regulates the expression of canonical EMT-inducing transcription factors, including Snail1/2, Slug, ZEB1, and Twist, in various malignancies [104]. YAP/TAZ knockdown has been shown to reverse cancer cell mesenchymal morphology [105]. Overall, these findings suggest that the Hippo pathway is involved in the activation and progression of the EMT processes. hnRNP-K is an RNA-binding protein (RBP) that belongs to the heterogeneous nuclear ribonucleoproteins (hnRNPs) family [106]. It has been reported that LINC01413 promoted EMT via a ZEB1-mediated mechanism in CRC. LINC01413/hnRNP-K/YAP1/TAZ1 axis prevented YAP1 and TAZ1 phosphorylations and thereby suppressing their degradation that resulted in increased nuclear translocation of TAZ1 and YAP1 in CRC cells. LINC01413 promoted ZEB1-mediated growth and metastasis of CRC cells via the LINC01413/hnRNP-K/YAP1/TAZ1 axis [107]. CASC21 has an oncogenic role in the proliferation and invasion of CRC cells. It has been reported that CASC21 acts as a ceRNA by miR-7-5p sponging to modulate YAP1 expression in CRC. CASC21 knockdown suppressed CRC cell proliferation, migration, and invasion while induced apoptosis via the Bcl-2/Bax axis. CASC21 knock down prevented EMT in CRC cells by CDH1 up regulation while Snail and Twist down regulations [108]. A MIR4435-2HG up regulation was observed in CRC tissue compared with normal samples that was correlated with poor prognosis. MIR4435-2HG knockdown reduced CRC cell proliferation, EMT, and migration by YAP1, VIM, Snail, and Twist down regulations while CDH1 up regulation. MIR4435-2HG up regulated YAP1 via miR-206 targeting [109]. ERG is a developmental transcription factor belongs to the external transcribed spacer (ETS) family [110]. This protein family is primarily engaged in the modulation of Hippo signaling [111]. Reduced levels of WWC2 have been demonstrated to play a crucial role in the clinicopathological characteristics of advanced hepatocellular carcinoma [112]. Inhibition of LINC00460 expression reduced the CRC cell proliferation, invasion, and EMT due to the elevated levels of WWC2 via ERG. LINC00460 down regulated WWC2 to promote EMT and metastasis in CRC cells [113].

## Polycomb repressive complex

Poly comb repressive complex (PRC) as a histone methyltransferase methylates H3K27me3 that is a hallmark of transcriptionally inactive chromatin. It is involved in cellular differentiation, early embryonic development, X chromosome inactivation, DNA damage response, and imprinting. Aberrant PRC2 expression and mutation have been reported in various human cancers [114, 115]. As the catalytic subunit of PRC2, EZH2 catalyzes the H3K27 methylation via an evolutionarily conserved histone methyltransferase domain [116]. It inhibits E-cadherin during EMT processes that results in tumor growth [117]. MALAT1 is an oncogenic lncRNA in non–small cell lung cancer via promoting cell invasion [118]. It binds directly to Polycomb 2 (PC2) subunit of the PRC1 [119]. MALAT1 was significantly up regulated in oxaliplatin resistant HT29 cells compared with sensitive HT29 cells. MALAT1 knockdown reversed the mesenchymal-like transition in both oxaliplatin resistant HT29 and sensitive HT29 cells. There was an inverse correlation between the levels of MALAT1 and CDH1 expressions in CRC cells and tissues. EZH2 up regulation was also correlated with advanced stages and reduced overall survival. MALAT1 sponged miR-218 to promote oxaliplatin-induced EMT via binding to EZH2 [120]. The EPH-receptor belongs to the protein-tyrosine kinases family that has a key role in cell–cell interactions by starting a specific bilateral signal transduction pathway between cells expressing Ephs and ephrins. Role of Ephs and ephrins in tumor formation and metastasis has been shown in a wide variety of human malignancies [121, 122]. SNHG14 promoted CRC progression by suppressing EPHA7 and up regulating EZH2 through recruitment of FUS and miR-186-5p sponging [123].

Histone acetylation, which is regulated by histone deacetylases (HDACs), is essential during tumor progression [124, 125]. Snail mediates the recruitment of the Sin3A/HDAC1/2 complex into the CDH1 promoter, where it inhibits CDH1 expression and enhances pancreatic cancer metastasis [126]. The interaction of EZH2 with Snail/HDAC1/HDAC2 complex was discovered to be required for the function of this complex [127]. Snail is a pivotal inducer of EMT process that requires HOTAIR to recruit the EZH2 that suppress the epithelial target genes. A HOTAIR deletion mutant form (HOTAIR-sbid) was designed without EZH2-binding domain while snail binding domain. It promoted H3K27me3/EZH2-mediated inhibition of Snail target genes that resulted in reduced cell migration [128]. Zinc finger E-box binding homeobox 1 (ZEB1) can also recruit HDAC2 to the CDH1 promoter region. It has been reported that there was HDAC2 up regulation in liver metastatic CRC compared with non-metastatic tumors. Up regulation of HDAC2 was associated with liver metastases and advanced T stages. Patients with increased HDAC2 expression had also a worse overall survival rate. The findings suggested that aberrant HDAC2 expression could be correlated with CRC metastases to the liver. Decreased HDAC2 levels have been found to reduce the migratory and invasive abilities of HCT116 cells through the EMT process. ENSG00000274093.1 enhanced tumor cell migration, invasion, and EMT by acting as a scaffold for HDAC2/HDAC1/EZH2 [129]. There were XIST up regulations in CRC tissues and cells that were correlated with a higher metastatic capacity. XIST promoted CRC cells migration and EMT via miR-137 sponging that resulted in EZH2 up regulation [130]. SNHG6 and EZH2 mRNA levels were increased in CRC tissues and cell lines, while miR-26a was down regulated. SNHG6 knock down also inhibited CRC cell invasion and EMT processes through miR-26a sponging and EZH2 regulation [131]. There was significant DUXAP8 up regulation in CRC tissues. DUXAP8 inhibited CDH1 expression while increased N-cadherin, vimentin, and Snail expressions. DUXAP8 promoted CRC cell proliferation, invasion, and EMT process, while suppressed apoptosis. Moreover, by interacting with EZH2 and H3K27me3, DUXAP8 inhibited E-cadherin expression to promote EMT during CRC progression [132].

## EMT-related transcription factors

**Figure.3.**
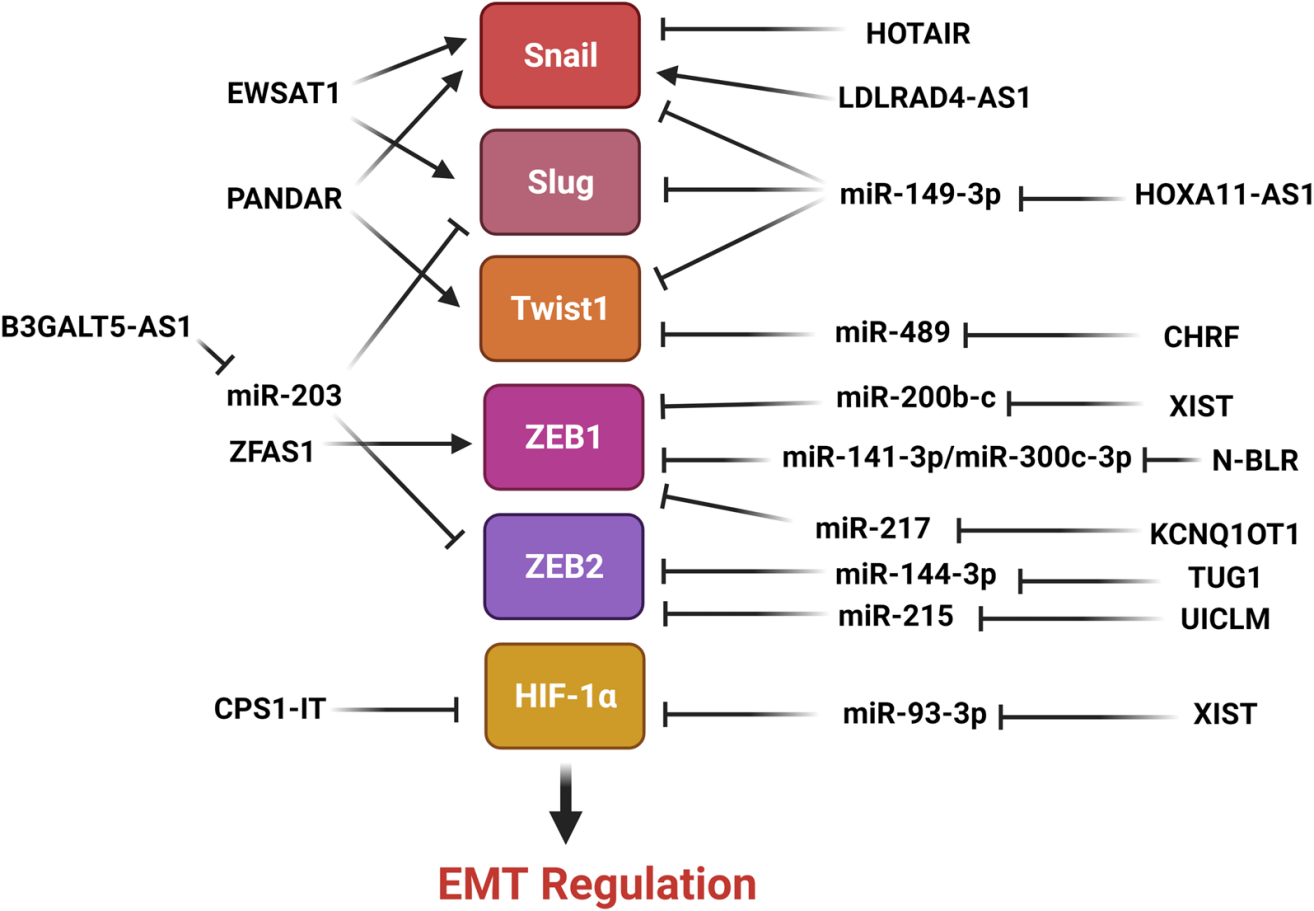
Role of lncRNAs in regulation of EMT-related transcription factors in CRC. Snail/Slug were inhibited by miR-149-3p and HOTAIR, while induced by LDLRAD4-AS1, EWSAT1, and PANDAR. Twist1 was activated by PANDAR, while suppressed by miR-149-3p and miR-489. ZEB1/2 was mainly inhibited by various microRNAs such as miR-217, miR-215, and miR-203. HIF-1α was also suppressed by miR-93-3p and CPS1-IT. (Created with www.BioRender.com)

EMT process is regulated by a series of specific transcription factors that are activated in downstream of various signaling pathways (Fig. 3). Slug is an essential EMT-related transcription factor that enhances tumor cell survival, invasion, and metastasis during cancer progression [133]. It has a limited half-life and its biological activities are dependent on its stability and total cellular concentration. Slug stability is preserved by a variety of processes. Slug degradation is mediated by MDM2 as an E3 ligase that binds to p53 through the UPP [134]. The stimulation of EMT by B3GALT5-AS1 through suppression of miR-203 has been identified as a unique regulatory axis during CRC liver metastasis. B3GALT5-AS1 inhibited miR-203 to increase ZEB2 and Slug which inhibited CRC liver metastasis [135]. Snail is a pivotal transcription factor during tumor cell invasion via the regulation of the EMT process [136]. By binding to E-box in the E-cadherin promoter, Snail was shown to suppress the E-cadherin expression, which is essential for cell polarity and epithelial differentiation [137]. Hepatocyte nuclear factor 4 alpha (HNF4α) belongs to the nuclear hormone receptor superfamily. HNF4α has been shown to operate as a tumor suppressor in hepatic cancer as a target gene of Snail [138]. It has been observed that HOTAIR knockdown inhibited the survival and metastasis of CRC cell lines in vitro, as well as the carcinogenic, migratory, and invasive abilities of CRC cells in vivo. HOTAIR induced EMT process and cell invasion via SNAIL recruitment and HNF4α down regulation in CRC cells [139]. HOTAIR up regulations were observed in tumor tissues in comparison with normal margins that were significantly correlated with lymph node involvement, histological grade, invasion depth, and advance stage. Patients with higher levels of HOTAIR expression exhibited a higher risk of recurrence, shorter metastasis-free survival, and overall survival rates than those with lower levels of HOTAIR expression. HOTAIR knockdown up regulated CDH1 while down regulated vimentin and MMP-9. Therefore, HOTAIR enhanced CRC invasion and EMT process [140]. There was HOXA11-AS up regulation in CRC tissues that was positively correlated with more aggressive clinicopathological characteristics. CRC patients with increased HOXA11-AS protein levels had a lower overall survival rate, larger tumor size, more recurrent metastasis, and advanced TNM stage. MiR-149-3p up regulated E-cadherin while down regulated Slug, Snail, and Twist. HOXA11-AS sponged miR-149-3p to promote EMT and metastasis in CRC cells [141]. PANDAR is an lncRNA that promotes metastasis and chromosomal instability in several malignancies. PANDAR expression was significantly up regulated in CRC tissues compared with normal margins. PANDAR up regulation was also positively correlated with depth of invasion, tumor size, tumor stage, histological differentiation, and lymph node metastasis. PANDAR down regulation suppressed proliferation, migration, invasion, and EMT while induced cell cycle arrest and apoptosis in CRC cells. PANDAR down regulation significantly reduced the levels of Snail and Twist expressions whereas up regulated the CDH1 [142]. It has been reported that EWSAT1 knocked down reduced cell proliferation, invasion, and EMT processes in CRC cells. EWSAT1 up regulation was correlated with depth of invasion, node metastasis, and TNM stage in CRC patients. Patients with EWSAT1 up regulation had lower overall survival rates compared with down regulated cases. EWSAT1 knockdown inhibited EMT in CRC cells by suppressing Snail and Slug while enhancing E-cadherin expression [143]. There were correlations between LDLRAD4-AS1 up regulation and clinicopathological features including advanced stage, lymph node metastasis, tumor size, and poor prognosis. LDLRAD4-AS1 reduced the levels of LDLRAD4 expression that resulted in Snail up regulation and E-cadherin down regulation in CRC [144]. There was miR-489 down regulation in CRC tissues compared with normal margins which was correlated with poor prognosis and tumor stage. MiR-489 inhibited CRC cell invasion and EMT process. MiR-489 down regulation induced by CHRF enhanced metastasis and EMT in CRC cells via TWIST1 targeting [145].

Histone deacetylases (HDACs) inhibit transcription by removing acetyl groups from lysine residues in histones. HDACs deacetylate non-histone and histone proteins. Several malignancies have been associated with abnormal expression of classical (class I, II, and IV) HDACs [146]. HDAC1 facilitates EMT in gallbladder cancer by interacting with TCF12 [147]. The recruitment of HDAC1 and HDAC2 by the transcriptional repressor ZEB1 results in CDH1 down regulation in pancreatic cancer [148]. SIRT1 cooperated with ZEB1 to promote EMT and increase prostate cancer cell invasion [149]. It has been observed that HDAC2 down regulation was correlated with low survival rates in CRC patients. HDAC2 inhibited EMT and CRC metastases via suppressing the H19 and MMP14. H19 enhanced EMT and CRC metastases via miR-22-3P sponging and MMP14 up regulation [150]. N-BLR up regulation was correlated with lower levels of miR-141-3p and miR-200c-3p expression and higher levels of ZEB1 expression. N-BLR up regulated ZEB1 to promote EMT characteristics by E-cadherin down regulation and vimentin up regulation. N-BLR was correlated with miR-200c-3p under expression and XIAP over expression [151]. KCNQ1OT1 is involved in suppression of various genes via interacting with chromatin and forming a specific chromatin conformation; eventually, chromatin and DNA-modifying proteins are recruited to complete the process [152, 153]. KCNQ1OT1 down regulation has been shown to diminish CRC cell proliferation, invasion, and EMT. KCNQ1OT1 promoted CRC progression by sponging miR-217 to increase ZEB1 expression [154]. It has been reported that there was ZFAS1 up regulation in colon cancer tissues and cell lines which was associated with adverse prognosis. ZFAS1 knocked down increased the E-cadherin and ZO-1 expressions, while down regulated the vimentin and N-cadherin. ZFAS1 might acted as an oncogene via regulating ZEB1 to promote EMT [155]. XIST up regulation was observed in CRC tissues compared to normal margins that was correlated with tumor size, lymph node invasion, and clinical stage. XIST up regulation was also associated with a lower overall survival rate, and thus it could be suggested as a prognostic marker for CRC patients. XIST down regulation inhibited cell invasion, proliferation, and EMT. It regulated ZEB1 expression by miR-200b-3p sponging [156]. XIST regulated WEE1 by miR-125b-2-3p sponging. XIST-miR-125b-2-3p-WEE1 modulated CRC growth, metastasis, and chemo resistance in CRC. MiR-125b-2-3p expression was significantly reduced in advanced stage CRC samples and predicted sensitivity to chemotherapy as well as prognosis in CRC cases [157]. TUG1 was significantly up regulated in CRC tissues and LoVo cells. TUG1 down regulation substantially suppressed ZEB2 expression, whereas transfection of the miR-138-5p-inhibitor reversed this suppression. TUG1 silencing and miR-138-5p up regulation significantly suppressed cell proliferation and invasion, decreased apoptosis-related protein β-catenin level, whereas elevated Bax and Caspase-3 levels. TUG1 down regulation dramatically suppressed ZEB2 expression in CRC and hampered CRC cell proliferation and EMT, whereas miR-144-3p up regulation reversed the suppression of EMT induced by TUG1 down regulation [158]. Liver is the most frequent distant metastatic site in CRC. About 25% of CRC patients exhibited synchronous liver metastases at the time of diagnosis, and approximately half of CRC patients had metachronous liver metastases three years following treatment [159]. The majority of colon cancer patients with liver metastases have poor prognosis [160]. It has been observed that UICLM induced CRC liver metastasis through miR-215 sponging that up regulated ZEB2 [161].

Nuclear HMGB2 facilitates EMT by interacting with OCT4 that enhances the formation of particular transcriptional complexes [162]. OCT4 overexpression is associated with reduced CDH1 expression and elevated levels of vimentin expression [163]. Hence, nuclear HMGB2 physically interacts with OCT4 to induce EMT and promote metastasis in CRC [163, 164]. It has been shown that Lnc-CRCMSL suppressed EMT in CRC cells via binding to HMGB2. Lnc-CRCMSL is a metastasis suppressor gene in CRC that modulates the nucleocytoplasmic shuttling of HMGB2. Although, lnc-CRCMSL expression was related to a favorable survival prognosis in CRC patients, it was inversely associated with higher clinical stages. CRCMSL inhibited EMT and metastasis of CRC via maintaining HMGB2 cytoplasmic localization. HMGB2 was retained in the cytoplasm by CRCMSL, which inhibited EMT and metastasis by interrupting the interaction of HMGB2 and OCT4 [165].

Hypoxia is a prevalent event in the microenvironment of solid tumors. Tumor cells have a high proliferation rate, and their oxygen and nutrient supply is considerably lower than that of normal cells. Aberrant vascular system structure and function also result in insufficient blood circulation, which fails to provide tumor cell oxygen and nutrient requirements following the formation of a hypoxic microenvironment [166, 167]. Hypoxia-inducible factor-1 (HIF-1) is a critical heterodimeric transcription factor that acts as a crucial regulator of oxygen homeostasis [168]. HIF-1α is involved in majority of human malignancies [169–171]. Hypoxia increased tumor cell invasion and metastasis by inducing autophagy via the HIF-1α signaling pathway. Autophagy is a vital intracellular process for cytoplasmic material degradation and recycling, which are the key to maintain cellular biosynthesis [172]. It has been reported that CPS1-IT inhibited metastasis and EMT by inactivating HIF-1α and, thus, suppressing hypoxia-induced autophagy in CRC [173]. There were significant XIST up regulations in CRC tissues compared with normal margins that was positively associated with TNM stage. XIST promoted the CRC progression via miR-93-5p targeting that resulted in HIF-1A/AXL signaling pathway regulation [174].

## Conclusions

Molecular mechanisms involved in EMT process are key targets for development of novel therapeutic modalities to suppress tumor metastasis. LncRNAs regulate epithelial-mesenchymal plasticity through affecting the miRNAs and protein expression profiles. Therefore, targeting the lncRNA and miRNA interactions can be an efficient way to inhibit the EMT and tumor progression in early stage carcinomas. However, targeting the EMT related lncRNA/miRNA axis is not a reliable therapeutic method for the patients with advanced stage and metastatic tumors. A variety of approaches such as RNA interference, Antisense oligonucleotides, and Aptamers are under assessments for lncRNAs. However, these methods are faced with the lack of efficient delivery methods. Although, further investigations are required for safe and efficient delivery method, some of them are currently used in clinical trial studies. It has been reported that lncRNAs mainly function as the inducers of EMT process in CRC through regulation of EMT-related transcription factors, PRC complex, and also signaling pathways including WNT, NOTCH, MAPK, and Hippo. Indeed, the EMT-related transcription factors are critical direct or indirect targets of lncRNAs in regulation of EMT in CRC in which they can be affected directly or in downstream of the mentioned signaling pathways.

## Data Availability

The datasets used and/or analyzed during the current study are available from the corresponding author on reasonable request.

## Abbreviations

CRC: Colorectal cancer
EMT: Epithelial-mesenchymal transition
lncRNAs: Long non-coding RNAs
PRC: Poly comb repressive complex
miRNAs: MicroRNAs
tRNA: Transfer RNA
eRNAs: Enhancer ncRNAs
ceRNA: Competing endogenous RNA
CK1: Casein kinase 1
DSL: Delta/Serrate/Lag2
NICD: Intracellular domain of the notch protein
SLC25A25: Solute carrier family 25 member 25
CaMC: Ca2 + -regulated mitochondrial carrier
MMP-14: Matrix metalloproteinase-14
MAPKAPK2: MAPK-activated protein kinase-2
MCP-1: Monocyte chemoattractant protein 1
TGF-β: Transforming growth factor-Beta
R-Smad: Receptor-regulated Smad
I-Smad: Inhibitory Smad
Co-Smad: Co-mediator Smad
SBE: Smad binding element
NMD: Nonsense-mediated mRNA decay
YBX1: Y-box binding protein 1
RBP: RNA-binding protein
hnRNPs: Heterogeneous nuclear ribonucleoproteins
ETS: External transcribed spacer
PRC: Poly comb repressive complex
PC2: Polycomb 2
HDACs: Histone deacetylases
ZEB1: Zinc finger E-box binding homeobox 1
HNF4α: Hepatocyte nuclear factor 4 alpha
HIF-1: Hypoxia-inducible factor-1
GSK3: Glycogen synthase kinase 3
APC: Adenomatosis polyposis coli
